# *CCND1* Amplification Profiling Identifies a Subtype of Melanoma Associated With Poor Survival and an Immunosuppressive Tumor Microenvironment

**DOI:** 10.3389/fimmu.2022.725679

**Published:** 2022-07-01

**Authors:** Jun Liu, Jing Lin, Xuefeng Wang, Xiaobin Zheng, Xuan Gao, Yingying Huang, Gang Chen, Jiani Xiong, Bin Lan, Chuanben Chen, Lu Si, Yu Chen

**Affiliations:** ^1^ Department of Medical Oncology, Fujian Medical University Cancer Hospital and Fujian Cancer Hospital, Fuzhou, China; ^2^ Cancer Bio-immunotherapy Center, Fujian Medical University Cancer Hospital and Fujian Cancer Hospital, Fuzhou, China; ^3^ Fujian Provincial Key Laboratory of Translational Cancer Medicine, Fujian Medical University Cancer Hospital and Fujian Cancer Hospital , Fuzhou, China; ^4^ The Third Affiliated Hospital of Soochow University and State Key Laboratory of Radiation Medicine and Protection, Institutes for Translational Medicine, Soochow University, Suzhou, China; ^5^ College of Clinical Medicine for Oncology, Fujian Medical University, Fuzhou, China; ^6^ Geneplus-Beijing, Beijing, China; ^7^ Department of Nasopharyngeal Carcinoma, Sun Yat-sen University Cancer Center, The State Key Laboratory of Oncology in South China, Collaborative Innovation Center for Cancer Center, Guangzhou, China; ^8^ Department of Pathology, Fujian Medical University Cancer Hospital and Fujian Cancer Hospital, Fuzhou, China; ^9^ Shanghai Center for Systems Biomedicine Research, Shanghai Jiao Tong University, Shanghai, China; ^10^ Department of Radiation Oncology, Fujian Medical University Cancer Hospital and Fujian Cancer Hospital, Fuzhou, China; ^11^ Department of Renal Cancer and Melanoma, Peking University Cancer Hospital and Institute, Beijing, China

**Keywords:** *CCND1* amplification, melanoma, poor survival, immune checkpoint inhibitors, tumor mutation burden, tumor microenvironment, oxidative and lipid metabolism signaling pathway

## Abstract

**Background:**

Although melanoma is generally regarded as an immunogenic cancer that will respond to immune checkpoint inhibitors (ICIs), melanomas with *CCND1* amplification respond poorly to these therapies. Further understanding how *CCND1* amplification impacts the effectiveness of ICI therapy is important for the design of future clinical trials.

**Methods:**

We used data from tumor samples taken from Chinese patients with melanoma analyzed at the Geneplus Institute (*n*=302), as well as data from the Cancer Genome Atlas (TCGA) database (*n*=367) and the Memorial Sloan Kettering Cancer Center (MSKCC) database (*n*=350) to estimate the prevalence of *CCND1* amplification in melanoma, interrogate the relationship between *CCND1* amplification and survival in patients with melanoma, and explore the molecular mechanisms of *CCND1* amplification. We also established a murine model of melanoma harboring *CCND1* amplification and utilized RNA-seq to verify the findings from human tissue samples.

**Results:**

Data from all three sources revealed a low frequency of *CCND1* amplification co-occurring with *BRAF V600*, *NRAS*, *NF1*, and *KIT* mutations. Data from TCGA did not show a statistically significant correlation between *CCND1* amplification and prognosis, irrespective of ICI use. In contrast, the MSKCC cohort showed that *CCND1* amplification was an unfavorable prognostic factor for patients with melanoma, especially for patients who received ICIs and had a high tumor mutation burden (TMB). The TCGA data showed that *CCND1* amplification was associated with a higher proportion of immunosuppressive cells (Treg cells and M2 macrophages) and a lower proportion of immune boosting cells (follicular helper T cells naïve B cells, CD8^+^ T cells). Murine models supported the association between a suppressive immune microenvironment and *CCND1* amplification; tumors with *CCND1* amplification had reduced mRNA expression of CD8, Gzm, B2m and Tap1, significantly higher proportions of resting CD4 memory T cells and significantly lower proportions of plasma cells, CD8 T cells, and T follicular helper cells. Furthermore, a Gene Set Enrichment Analysis (GSEA) analysis of data from the TCGA database suggested that signaling pathways involved in oxidative phosphorylation, reactive oxygen species, adipogenesis, fatty acid metabolism, DNA repair, and Myc targets were differentially enriched in melanoma tumors with *CCND1* amplification. Finally, we observed a notable reduction in levels of angiogenesis-related molecules (encoded by *HIF1A, VEGFA, VEGFR1, FGF2, FGFR1, FGFR4, HGF*, *PDGFA*, *PDGFRA*, *ANGPT1*, and *ANGPT2*) in a high *CCND1* amplification group from the TCGA database.

**Conclusions:**

Melanoma with *CCND1* amplification is an independent genomic subtype associated with a poor prognosis, an immunosuppressive TME, activated oxidative and lipid metabolism, and down-regulated angiogenesis. Therefore, avoiding ICIs and antiangiogenic agents, while employing CDK4/6 inhibitors alone or in combination with ICIs, and targeting oxidative and lipid metabolism pathways, may be effective therapeutic strategies for melanoma patients harboring *CCND1* amplification.

## Introduction

Melanoma is one of the deadliest cancers due to its high recurrence rate and high metastatic potential (1–4). Although the incidence of melanoma is rising worldwide (5, 6), melanoma tumors are generally characterized by high immunogenicity, which theoretically makes them good candidates for immunotherapy. However, more than 50% of patients with melanoma do not respond to treatment with immune checkpoint inhibitors (ICIs; inhibitors of programmed death-1/programmed death ligand-1 [PD-1/PD-L1]) (2, 7). Therefore, while immunotherapy represents an important advancement in the treatment of melanoma, there remains a need to develop new effective therapies and further understand the mechanisms behind the high rate of non-response to ICIs.

Melanoma tumors are broadly categorized into four subtypes: acral, mucosal, non-acral cutaneous, and unknown primary melanoma, based on their clinical and pathological features (8, 9). These different subtypes are associated with differential response rates to ICIs (10–12). Recently, advances in molecular biology have revealed that melanomas are also genetically heterogeneous. In this regard, the Cancer Genome Atlas (TCGA) program has established a framework of genomic classification for cutaneous melanomas as BRAF, NRAS, NF1, and Triple Wild-Type (WT), and serves as a guide when making decisions about therapy (13).

The classification of melanomas using the TCGA framework facilitates the discovery of common characteristics shared within the genetic subsets, therefore providing signposts for prognosis and therapy. This deeper understanding of melanoma at the level of the molecular and immune microenvironment has been successfully leveraged to identify novel treatment targets and strategies following a personalized medicine approach. For example, melanomas harboring mutations of driver events in the MAPK pathway (BRAF, NRAS, NF1, KIT, and so on) have been shown to respond well to MEK/MAPK inhibitors (2, 14–26). Furthermore, approximately half of melanomas are reported as an “immune” subtype and around one-third of patients with this subtype respond to treatment with ICIs (13, 27–29). Although these previous findings represent significant advances in melanoma treatment, they also suggest that many patients have melanomas that are challenging to treat due to an absence of hot-spot mutations or resistance to ICIs. Therefore, the development of new and effective therapeutic strategies for patients without specific mutations or immunogenic tumors is an urgent unmet need.

We have previously reported findings from a sample of melanoma tumors that revealed significant enrichment for cyclin D1 (CCND1) amplification in patients with a Triple-WT genomic classification and these patients had a lower response rate to ICIs (13, 30). The product of the *CCND1* gene is a G1 phase cell cycle regulator and *CCND1* therefore acts as an oncogene (31, 32). In our early research on the characterization of *CCND1* amplification in pan-cancer (nine solid tumor types) using data from three databases (Geneplus, TCGA, and MSKCC) we confirmed that *CCND1* amplification is associated with a poor response to ICIs. We also found the overexpression of *VEGFA*, *HIF1A, PDGFA-D, FGF2, HGF*, and the activating pathways of epithelial mesenchymal transition, TGF-β signaling, and KRAS signaling may contribute to immunosuppression in *CCND1* amplified tumors (33). Given the unique properties and complexity of melanoma, it is essential to explore the *CCND1* amplification landscape specific to melanoma and identify unique pathopoiesis mechanisms. Building on our previous work, in the present study, we further investigated the prevalence of *CCND1* amplification and the relationship between *CCND1* amplification and survival in patients with melanoma, and explored the potential molecular signaling pathways implicated in melanoma with *CCND1* amplification.

## Methods

### Samples

Tumor tissue samples were obtained from 302 Chinese patients with melanoma and sent for next-generation sequencing (NGS) at Geneplus-Beijing (Beijing, China). Corresponding clinical data for these patients were collected from our records. This study was approved by the Ethics Committee of Fujian Provincial Cancer Hospital. Written, informed consent was obtained from all participants before inclusion.

### DNA Extraction

Germline genomic DNA was extracted from peripheral blood lymphocytes and frozen tissue samples using the DNeasy Blood & Tissue Kit (Qiagen, Hilden, Germany). DNA from formalin-fixed, paraffin-embedded (FFPE) tissue samples was isolated using Maxwell^®^ 16 FFPE Plus LEV DNA Purification (Qiagen, Hilden, Germany Kit. catalog: AS1135) according to the manufacturer’s protocol.

### NGS and Analysis

NGS was carried out as previously reported (33). Illumina 2 × 75-bp paired-end reads on an Illumina HiSeq 3000 instrument and KAPA DNA Library Preparation Kit (Kapa Biosystems, Wilmington, MA, USA) were used for sequencing. Barcoded libraries were hybridized to a customized panel of 1021 genes. The libraries were sequenced to a uniform median depth (>500×) and assessed for somatic variants including single nucleotide variants (SNVs), small insertions and deletions (InDels), copy number alterations (CNA), and gene fusions/rearrangements. Contra (v2.0.8) was employed to identify CNAs (34). The CNA number was expressed as the ratio of adjusted depth between ctDNA and germline DNA, and was analyzed using FACETS with log2ratio thresholds of 0.848 and -0.515 for gain and loss, respectively (35). Specifically, for the *CCND1* gene, samples with chromosome 11q13.3 alterations were further reviewed for CNAs. We have uploaded the sequencing data of the geneplus cohort to the EMBL-EBI European Nucleotide Archive, the project: PRJEB50175; Analyses: ERZ4866806.

### Data From the TCGA and MSKCC Databases

Data from 367 tumor tissue samples from melanomas with CNAs included in the TCGA database were obtained from the Broad Institute Genomic Data Analysis Center (https://gdac.broadinstitute.org/). The TCGA cohort consisted of skin cutaneous melanomas (SKCM). Survival information and RSEM-normalized gene level data were also downloaded.

Data from 306 tumor tissue samples from melanomas with CNAs included in the MSKCC database and corresponding patient survival information were downloaded. A total of 231 patients who had received at least one dose of ICIs at MSKCC were included in a MSKCC-immunotherapy (MSKCC-IO) cohort which is a subset of MSKCC cohort. For these patients, overall survival (OS) was defined as the time from the date of the first infusion of any ICI to death from any cause.

### Database Analysis for *CCND1* and Tumor Mutation Burden

The FACETS algorithm was used to define CN changes, including putative biallelic CN amplification (+2) and putative biallelic neutral (0) for samples from patients in the MSKCC cohort (35). The total number of somatic mutations identified was normalized to the exonic coverage of the MSK-IMPACT panel in megabases. Cases in the top twentieth, fortieth, sixtieth, and eightieth percentiles of TMB were identified. Cases within the top twentieth percentile of TMB were categorized as the TMB-High group (*n*=51).

### Tumor Purity Estimate and Infiltrating Immune Cells Inference

The ESTIMATE tool was used to analyze immune components and overall stroma in the TCGA cohort (36). The CIBERSORT algorithm was employed to profile the infiltration levels of 22 immune cell populations (37).

### Gene Set Enrichment Analysis

Gene Set Enrichment Analysis software version 3.0 (Broad Institute) was used to verify the activated signaling pathways in the *CCND1* amplification group compared to the *CCND1* neutral group in the TCGA melanoma cohort.

### Lentivirus-Mediated Overexpression of *CCND1* in the B16 Melanoma Cell Line

For overexpression of CCND1, mRNA-derived Ccnd1 cDNA sequence were cloned under control of the EF1α promoter in the lentiviral vector pCDH. The generation of lentivirus vectors was performed by co-transfecting pCDH carrying the expression cassette with helper plasmids pMD2.g and psPAX2 into HEK 293T cells using Lipofectamine 3000 reagent (Invitrogen Life Technology, Waltham, MA). The viral supernatant was collected 48 hours after transfection, and viral titers were determined by transducing HeLa cells at serial dilutions and analyzing the GFP expression using flow cytometry. Cells at 50–70% confluency were infected with viral supernatants containing 10 mg/ml Polybrene for 24 hours, after which fresh medium was added to the infected cells, which were later selected with puromycin.

### Mouse Xenograft Model and RNA-Seq Analysis

Tumor models were established in female C57BL/6 mice aged 6 weeks. Animal care and handling procedures were performed in accordance with the Guide for the Care and Use of Laboratory Animals, and the animal study protocol was approved by the Institutional Animal Care and Use Committee of Fujian Medical University. *CCND1* amplification B16 cells or control cells (5×10^5^ cells for each mouse) were injected subcutaneously into the backs of mice. Tumor volumes were monitored and recorded every three days. The tumor volumes were estimated using the following formula: 0.5 × length×width^2^. RNA-Seq experiments were used to investigate the transcriptional profiles, and it was did by Shanghai KangChen Biotech Company, Shanghai, China (http://www.kangchen.com.cn). Corresponding GEO accession numbers were GSE180327.

### Statistical Analysis

Two-tailed, unpaired *t-*tests were used to compare intergroup differences in variables. Kaplan-Meier survival curves and multivariate Cox regression analyses were used to analyze associations between *CCND1* status and survival. Log-rank tests were used to evaluate the statistical significance of differences between survival curves stratified by TMB. The SPSS statistical software version 23.0 (SPSS), Prism analysis and graphic software version 8.0.1 (GraphPad) and R Foundation for Statistics Computing, R script (v3.6.0) were used for difference analyses. Statistical significance was defined as *P*<0.05.

## Results

### Prevalence of *CCND1* Amplification in Melanoma

**Figure 1:**
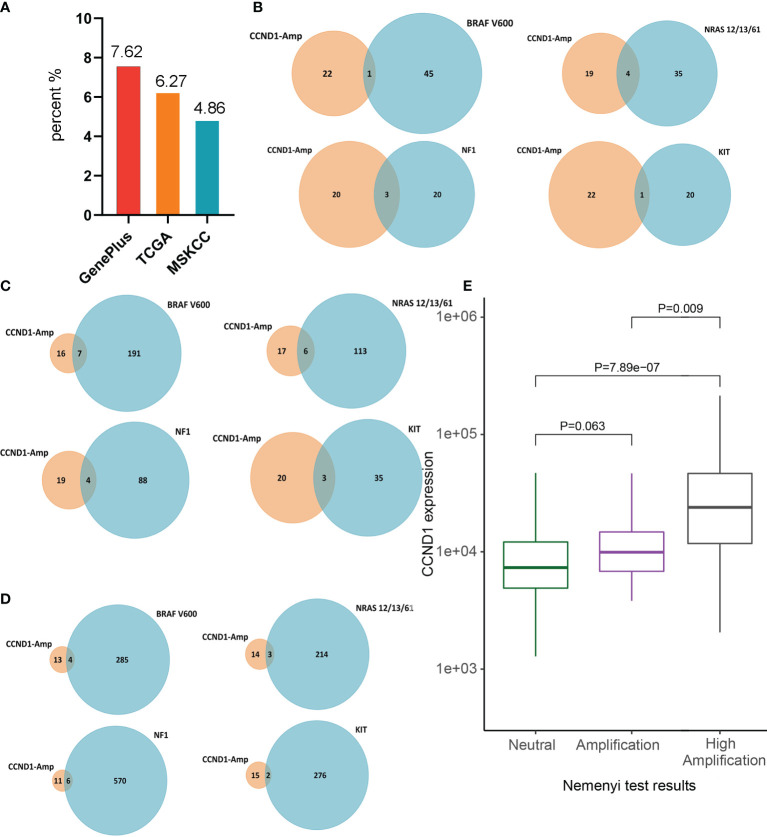
Prevalence of *CCND1* amplification in melanoma. **(A)** The prevalence of *CCND1* amplification calculated using data from Chinese patients with melanoma analyzed by Geneplus (*n*=302) and using data from the TCGA (*n*=367) and MSKCC (*n*=350) databases. **(B)** The frequency of co-occurrence of *CCND1* amplification with *BRAF V600*, *NRAS*, *NF1*, *KIT* in samples from Chinese patients with melanoma analyzed by Geneplus. The frequency of co-occurrence of *CCND1* amplification with *BRAF V600*, *NRAS*, *NF1*, *KIT* in samples from the **(C)** TCGA and **(D)** MSKCC databases. **(E)** *CCND1* mRNA expression in the *CCND1* Neutral group (*n*=191), Amplification group (*n*=46) and High Amplification group (*n*=23) in samples from the TCGA database. MSKCC, the Memorial Sloan Kettering Cancer Center; TCGA, The Cancer Genome Atlas.

Among the 302 Chinese melanoma patients whose tumor tissue underwent targeted sequencing with a 1021-gene panel we found *CCND1* amplification in 7.62% of cases (23/302). Furthermore, among the cohorts from the TCGA and MSKCC databases, the incidences of *CCND1* amplification were 6.3% (23/367) and 4.9% (17/350), respectively (**Figure 1A**).

To characterize the molecular heterogeneity among the four genomic subtypes of cutaneous melanomas, the co-occurrence of *CCND1* amplification with major driver mutations (*BRAF V600*, *NRAS*, *NF1*, and *KIT*) was analyzed in the three cohorts. The *BRAF* mutation is the most common mutant gene in melanoma and co-occurred with *CCND1* amplification in 0.33% (1/302), 1.91% (7/367), and 1.14% (4/350) of tumor samples from the Geneplus, TCGA, and MSKCC cohorts, respectively. *RAS* is the second major mutant gene in melanoma and the co-occurrence frequency with *CCND1* amplification was 1.32% (4/302), 1.63% (6/367), and 0.86% (3/350) among the Geneplus, TCGA, and MSKCC cohorts, respectively. The third most frequently observed mutated gene in our analysis was *NF1*, which co-occurred with *CCND1* amplification in 0.99% (3/302), 1.09% (4/367), and 1.71% (6/350) of samples from the Geneplus, TCGA, and MSKCC cohorts, respectively. In addition, the *KIT* mutation co-occurred with *CCND1* amplification in 0.33% (1/302), 0.82% (3/367), and 0.57% (2/350) of tumor samples in the Geneplus, TCGA, and MSKCC cohorts, respectively (**Figures 1B–D**).

To investigate the relationship between *CCND1* amplification and *CCND1* mRNA expression, we stratified the 367 melanoma samples from TCGA based on *CCND1* amplification levels. We assigned each melanoma sample a *CCND1* amplification level score, defined as: Neutral group (*n*=191), Amplification group (+1; *n*=46), and High Amplification group (+2; *n*=23) (38, 39). Gene expression analysis showed that the *CCND1* High Amplification group expressed a significantly higher level of *CCND1* mRNA compared to the Neutral group (*P*=7.89e-07) and the Amplification group (*P*=0.009). The *CCND1* Amplification group exhibited higher *CCND1* mRNA expression than the Neutral group but the difference did not reach statistical significance (*P*=0.062) (**Figure 1E**). These data suggest that *CCND1* amplification level score +2 is the optimal cut-off to distinguish *CCND1* mRNA expression level.

### Association Between *CCND1* Amplification and Survival

**Figure 2:**
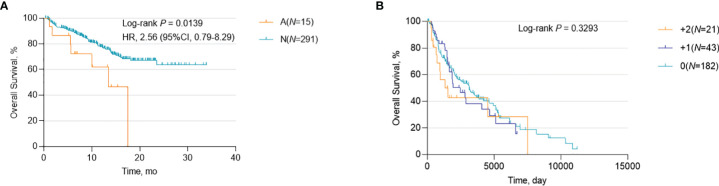
Association between survival and *CCND1* amplification in patients with melanoma. Kaplan-Meier survival analysis of melanoma patients, regardless of ICI use, calculated using data from **(A)** the MSKCC database (*n*=306) and **(B)** the TCGA database (*n*=246). ICI, immune checkpoint inhibitor; MSKCC, the Memorial Sloan Kettering Cancer Center; TCGA, The Cancer Genome Atlas.

To determine whether *CCND1* amplification can provide prognostic information for patients with melanoma, regardless of ICIs use, we investigated the association between *CCND1* amplification and OS curves using data from the TCGA and MSKCC databases. Data from the MSKCC database showed that *CCND1* amplification was associated with poor survival (*P*=0.0139) (**Figure 2A**), consistent with prior reports (33, 40). However, in contrast, data from the TCGA database showed no association between *CCND1* amplification and survival (*P*=0.3293) (**Figure 2B**). We further analyzed the copy number variation (CNV) of genes in 11q13.3 locus of 1105 samples from patients received ICI treatment in MSKCC-IO cohort. Genes in 11q13.3 locus include *CCND1*, *FGF3*, *FGF4* and *FGF19*. We calculated the co-amplificative frequency of *FGF3* (88.46%), *FGF4* (88.46%) and *FGF19* (88.46%). We did the univariate analyses in MSKCC-IO cohort. The result is displayed in **Table S1**. The result indicated that amplifications of *CCND1*, *FGF3*, *FGF4* and *FGF19* were all negative prognostic factors, with statistical significance. Therefore, co-amplified *FGF3*, *FGF4* and *FGF19* may also contribute to the worse outcome (41). PAK1 and GAB2 were well known regulators of the MAPK pathway and might participate in the regulation of melanoma development and response to therapies. We found that 56% had P*AK1* high amplification and 52% had *GAB2* high amplification among *CCND1* high amplification melanoma patients **Figure S1**, indicating that co-amplified *PAK1* and *GAB2* should also participated the worse outcome of melanoma.

### Association Between *CCND1* Amplification and Survival in Melanoma Patients After ICI Treatment

**Figure 3:**
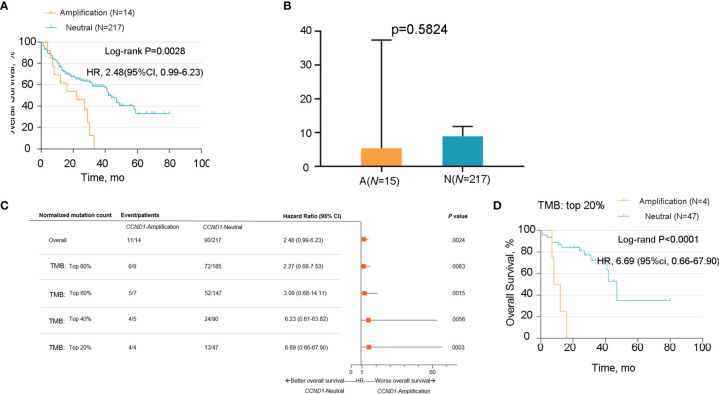
Association between survival and *CCND1* amplification in melanoma patients after treatment with ICIs in the MSKCC-IO cohort (*n*=231). **(A)** The relationship between survival and *CCND1* amplification in the MSKCC-IO cohort. **(B)** The correlation between TMB and *CCND1* amplification in samples from patients with melanoma included in the MSKCC-IO cohort (*n*=232). **(C)** Hazard ratio of *CCND1* status across melanoma patients with different level of TMB in the MSKCC-IO cohort (*n*=232). **(D)** Kaplan-Meier survival analysis of melanoma patients in the top 20% TMB within each histology in the MSKCC-IO TMB-high subgroup (*n*=51). ^a^The MSKCC-IO cohort included data from patients in the MSKCC database who had received at least one dose of ICIs. ICI, immune checkpoint inhibitor; MSKCC, the Memorial Sloan Kettering Cancer Center; TCGA, The Cancer Genome Atlas; TMB, tumor mutation burden.

A correlation between a high level of somatic copy number alterations (SCNAs) and poor survival after immunotherapy has been previously described in multiple cancer types (40–44). Our early pan-cancer study also reported that patients with *CCND1* amplification benefit less from ICIs (33). In this study, we used data from the MSKCC database to verify these previous results in melanoma patients who had received ICIs. We defined melanoma patients who had received MSKCC-IMPACT testing and at least one dose of ICIs as the MSKCC-IO cohort; the characteristics of the 14 patients with *CCND1* amplification in the MSKCC-IO cohort are shown in **Table S2**. Firstly, we assessed the relationship between *CCND1* amplification and OS in the MSKCC-IO cohort. As expected, melanoma patients with *CCND1* amplification showed significantly shorter OS than the *CCND1* neutral group (*P*=0.0029) (**Figure 3A**).

In light of the previously-reported correlation between TMB and immune activity in the tumor microenvironment (45, 46), we sought to determine whether there was a correlation between TMB and *CCND1* amplification in melanoma using data from the MSKCC-IO cohort. We compared the TMB between the *CCND1* Amplification and Neutral groups and found no significant difference (*P*=0.5824) (**Figure 3B**). We subsequently stratified the MSKCC IO-cohort based on the percentile of TMB, and the result showed that patients with melanoma harboring *CCND1* amplification did not benefit from ICIs regardless of TMB status (**Figure 3C**). A cut-off of the top 20% TMB values (3.6 muts/Mb) was selected to define the TMB-High population in this study as suggested by Samstein et al. (45). Stratified analysis of the TMB-High subgroup of the MSKCC cohort showed *CCND1* amplification was associated with poor survival (*P*<0.0001) (**Figure 3D**). Finally, we utilized a multivariable Cox proportional hazards model to derive a combined risk score. In this multivariable model, the contribution of *CCND1* amplification was significant (hazard ratio=2.439, *P*=0.006) (**Table S3**). Our data showed that the median OS in the *CCND1* amplification group was significantly shorter than for the non *CCND1* amplification group after adjustment for TMB, age, drug class of ICI, and the year that ICI treatment was initiated (*P*=0.006) (**Table S3**).

### Immune Landscape of the *CCND1* Amplification in Melanoma

**Figure 4:**
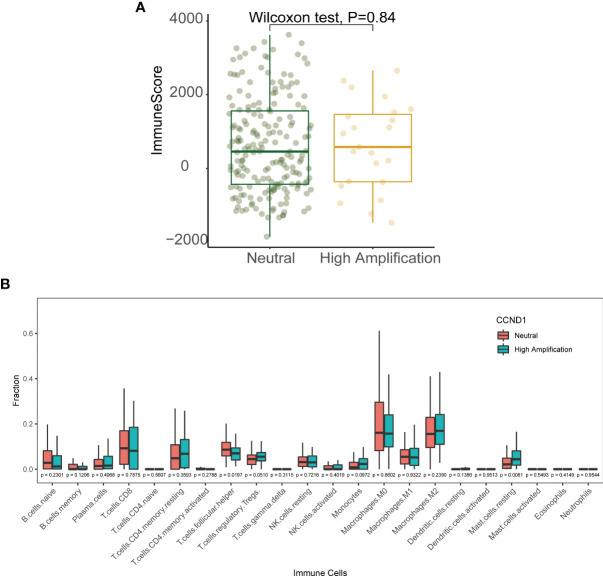
Immune landscape of the *CCND1* amplification in samples from patients with melanoma in the TCGA database. **(A)** The relationship between *CCND1* amplification status and infiltrating fraction of stromal and immune cells using ESTIMATE on data from the TCGA database (*n*=237). **(B)** The relationship between *CCND1* amplification status and immune infiltration of 22 immune cell subsets using CIBERSORT on data from the TCGA database (*n*=237). MSKCC, the Memorial Sloan Kettering Cancer Center; TCGA, The Cancer Genome Atlas.

Emerging evidence suggests that *CCND1* amplification is associated with tumor immunosuppression and inhibition of anti-tumor immune effector cells across multiple cancer types (33, 40). In the present study, we investigated the association between *CCND1* amplification and the tumor immune microenvironment in melanoma using data from the TCGA cohort. Firstly, we evaluated the relationship between *CCND1* amplification and the infiltrating fraction of stromal and immune cells using the ESTIMATE tool and did not observe any difference between patients with and without *CCND1* amplification (*P*=0.84) (**Figure 4A**). Next, we used CIBERSORT to evaluate the immune infiltration of 22 immune cell subsets whose expression was up-regulated or down-regulated in melanoma with *CCND1* amplification (**Figure 4B**). The *CCND1* High Amplification group had significantly higher proportions of Tregs (*P*=0.0510) and resting mast cells (*P*=0.0081), and significantly lower proportions of T follicular helper cells (*P*=0.019). However, no significantly higher proportion of immunosuppressive cells (e.g., M2 macrophages) and no significantly lower proportion of immunity boosting cells (e.g., naïve B cells, CD8^+^ T cells) was observed in the *CCND1* High Amplification group. Overall, there was a clear trend towards *CCND1* amplification promoting an immunosuppressive microenvironment. These results suggest that the TME of melanoma tumors with *CCND1* amplification is more inclined to be immunosuppressive and tumorigenic.

### Signaling Pathways and Angiogenesis Molecules Associated With *CCND1* Amplification in Melanoma

**Figure 5:**
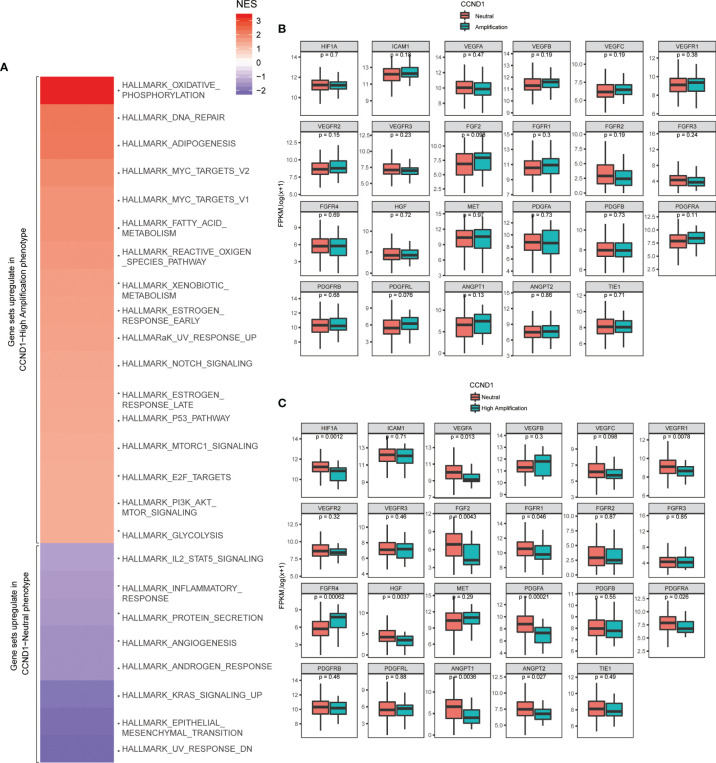
Identification of signaling pathways and angiogenesis molecules associated with *CCND1* amplification in patients with melanoma included in the TCGA database (*n*=237). **(A)** Activated signaling pathways in patients with melanoma from the TCGA database categorized into *CCND1* High Amplification and Neutral groups using GSEA analysis. Prevalence of TME-related angiogenesis molecules in patients with melanoma from the TCGA database categorized into **(B)** *CCND1* Amplification and Neutral groups and **(C)** High Amplification and Neutral groups. TCGA, The Cancer Genome Atlas; TME, tumor microenvironment.

To further verify signaling pathways activated in melanoma with *CCND1* amplification, we performed a GSEA comparing the *CCND1* Amplification and *CCND1* Neutral groups using data from TCGA. Gene sets were differentially enriched in the *CCND1* High Amplification group, as they were related to processes of oxidative metabolism and lipid metabolism, such as oxidative phosphorylation, reactive oxygen species, adipogenesis, fatty acid metabolism, DNA repair, and Myc targets (**Figure 5A**).

Finally, we analyzed the prevalence of TME-related angiogenesis molecules in melanoma with *CCND1* amplification using data from the TCGA database. There were no significant differences between the *CCND1* Amplification group and the *CCND1* Neutral group (**Figure 5B**). Overall, significant decreases in *HIF1A* (*P*=0.0012), *VEGFA* (*P*=0.013), *VEGFR1* (*P*=0.0078), *FGF2* (*P*=0.0043), *FGFR1* (*P*=0.046), *FGFR4* (*P*=0.00062), *HGF* (*P*=0.0037), *PDGFA* (*P*=0.00021), *PDGFRA* (*P*=0.026), *ANGPT1* (*P*=0.0036), and *ANGPT2* (*P*=0.027) were observed in the *CCND1* High Amplification group compared to the *CCND1* Neutral group (**Figure 5C**).

### Murine Models of *CCND1* Amplified Melanoma

**Figure 6:**
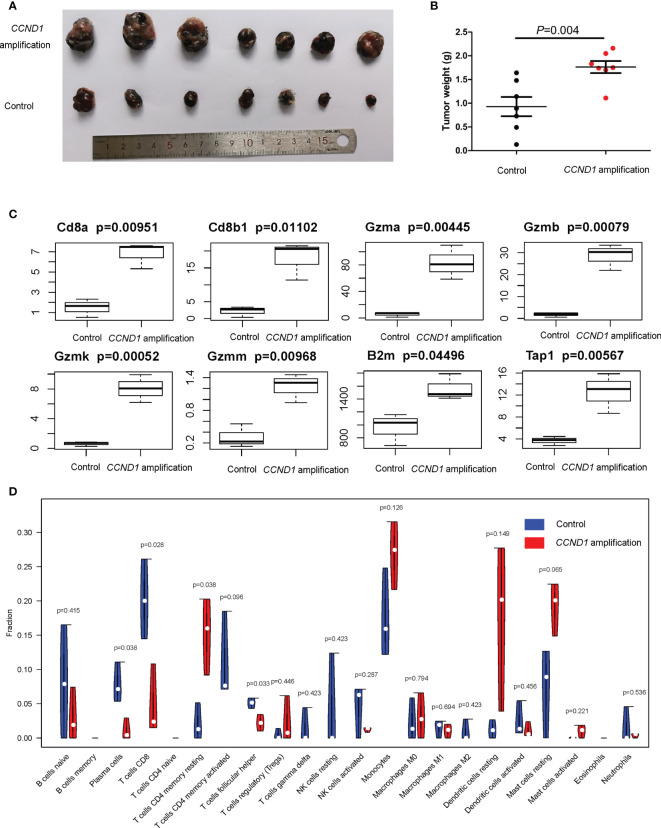
Mice models of melanoma with *CCND1* amplification interrogating the associated immune microenvironment. **(A)** Tumor appearance in each mouse was photographed. **(B)** The tumors were weighed 24 days after transplant. **(C)** Expression of CD8a, CD8b1, Gzma, Gzmb, Gzmk, Gzmm, B2m and Tap1 in *CCND1* amplification tumor tissues and controls measured using RNA-seq (*n*=3), error bars represent ± standard deviation. **(D)** The relationship between *CCND1* amplification status and immune infiltration of 22 immune cell subsets using the CIBERSORT (*n*=3).

In order to further investigate the immune microenvironment of *CCND1* amplification, we developed a murine model of melanoma with *CCND1* amplification. We subcutaneously injected *CCND1* amplified B16 cells and control B16 cells into C57 mice. After 3 weeks, we observed that the *CCND1* amplified B16 cells produced much larger tumors than those derived from the control B16 cells (**Figures 6A, B**). Furthermore, the RNA-seq results summarized in **Figure 6C** show that the mRNA expression of CD8, Gzm, B2m, and Tap1 were decreased in tissue samples from the tumors with *CCND1* amplification (*P*<0.05). Furthermore, we also used CIBERSORT to evaluate the infiltration of 22 immune cell subsets in mice tumor tissue samples. As shown in **Figure 6D**, the tumors with *CCND1* amplification had significantly higher proportions of resting CD4 memory T cells (*P*=0.0380), and significantly lower proportions of plasma cells (*P*=0.038), CD8^+^ T cells (*P*=0.028), and T follicular helper cells (*P*=0.0330). Although the higher proportion of immunosuppressive cells (e.g., Tregs and monocytes) and lower proportion of immunity boosting cells (e.g., naive B cells, activated CD4 memory T cells and activated NK cells) observed did not reach significance in the tumors with *CCND1* amplification versus controls, these results indicated that *CCND1* amplification promoted an immunosuppressive tendency in tumor microenvironment and consistent with the findings of our bioinformatic analysis.

## Discussion

In this study, we explored the profile of melanoma with *CCND1* amplification. Using data generated from melanoma tissue samples from Chinese patients, as well as the TCGA, and MSKCC databases, we have shown the following: 1) melanoma with *CCND1* amplification is an independent genomic subtype, 2) *CCND1* amplification is associated with poor prognosis in unselected melanoma patients as well as in melanoma patients treated with ICIs, and this association is stronger among patients with a high TMB, 3) *CCND1* amplification is related to an immunosuppressive TME, down-regulation of angiogenesis, and increased oxidative metabolism and lipid metabolism. Our findings support and provide an explanation for prior observations, that is despite melanoma being an immunogenic cancer type, ICIs have failed to demonstrate meaningful clinical benefits for treating melanoma tumors with *CCND1* amplification (30). We believe our results provide an important foundation for developing novel therapeutic strategies in this patient population.

*CCND1*, a G1 phase cell cycle regulator, is an oncogenic factor. Accumulating evidence shows that *CCND1* amplification is a possible risk factor for many cancers (31, 32). In a study which classed cutaneous melanomas into four genomic subtypes according to the pattern of the most prevalent significantly mutated genes, *CCND1* amplification was only significantly enriched in the Triple-WT subtype (13). This suggests that *CCND1* amplification is mutually exclusive with *BRAF*, *RAS*, and *NF1* mutations, which account for the three most common mutant genes observed in melanoma. Data from the present study also show a low frequency of co-occurrence of *CCND1* amplification with the above common mutations, supporting the opinion that *CCND1* amplification should be considered an independent genomic subtype in melanoma and may have unique biological characteristics.

The introduction of ICIs has greatly improved clinical outcomes for many patients with melanoma. However, it has been well documented that a high level of tumor SCNA correlates with worse patient survival and the magnitude of SCNA has an effect on the survival of patients receiving immunotherapy (40–44). In the present study, data from melanoma patients included in the TCGA database with different *CCND1* amplification status, irrespective of ICI treatment, did not show a statistically significant association between *CCND1* amplification levels and prognosis (**Figure 2B**). In contrast, analysis of a further data set from the MSKCC database found a significant association between *CCND1* amplification and worse survival (**Figure 2A**). In addition, for patients in the MSKCC cohort who received ICIs, *CCND1* amplification was also significantly associated with worse survival outcomes (**Figure 3A**), an observation that has been previously described (30, 33). There was a particularly notable association between patients with *CCND1* amplification and high TMB and poor survival outcomes following immunotherapy (**Figure 3E**), a result that opposes the popular concept that patients with high TMB are expected to benefit from ICIs. Our data demonstrate that *CCND1* amplification is a stronger prognostic factor for outcomes following immunotherapy in melanoma than TMB and indicate that *CCND1* amplification dramatically reduces patient survival regardless of the use of ICIs or TMB. Therefore, we conclude that ICIs may not be a suitable treatment for patients with melanoma harboring a *CCND1* amplification.

An improved understanding of the microenvironment of melanoma harboring *CCND1* amplification will support the development of new and effective therapies. Consistent with our prior findings, the present study showed that *CCND1* amplification was associated with higher proportions of Tregs and lower proportions of T follicular helper cells, CD8^+^ T cells, naïve B cells, and M2 macrophages, suggesting a trend toward an immunosuppressive immune microenvironment (33). Furthermore, we successfully established mice models of melanoma with a *CCND1* amplification which recreated a similar immunosuppressive microenvironment (**Figure 6**). Specifically, *CCND1* amplified tumor tissues exhibited decreased mRNA expression of CD8, Gzm, B2m and Tap1 and significantly lower proportions of CD8 T cell and T follicular helper cells. Taken together, the results from our bioinformatic analysis and animal experiments confirm that *CCND1* amplification facilitates an immunosuppressive immune microenvironment. There exist methodological limitations in characterizing TME. Our study is a preliminary investigation mainly focused on the predictive function of *CCND1* amplification in TME in the aspect of genome and transcriptome, so we employed ESTIMATE and CIBERSORT algorithms. The full implication of *CCND1* amplification requires in-depth studies, and we will perform more biological insights such as single cell RNA-seq or CyTOF to investigate the direct mechanism of *CCND1* amplification and primary immune resistance in future. Moreover, CDK4/6 inhibitors were recently reported to enhance the susceptibility of tumors to ICIs by suppressing Treg proliferation (47). Considering *CCND1* is an important driver of CDK4, CDK4/6 inhibitors as monotherapy or in combination with ICIs may represent a highly promising treatment for patients with melanoma harboring *CCND1* amplification (30, 48). Secondly, HIF1A, VEGFs, angiopoietin growth factors, MET, HGF, PDGFs and FGF2/FGFR2 are TME-related molecules (49) and previous studies had revealed that cyclin D1 may play a key role in the maintenance of VEGFs, and antisense to cyclin D1 could be useful for targeting both cancer cells and blood vessels in tumor (50), we analyzed the RNA-Seq data in TCGA focusing on above-mentioned single genes. In contrast to our previous findings in a TCGA pan-cancer cohort (33), patients with melanoma in the *CCND1* High Amplification group in the present study showed downregulation of many genes associated with angiogenesis (*HIF1A*, *VEGFs*, *VEGFRs*, *FGFs*, and *FGFRs*) (**Figure 5C**). This suggests that angiogenesis may not contribute to the initiation and progression of melanoma tumors harboring a *CCND1* amplification, and antiangiogenic agents may not be effective in this subset of patients.

Our signaling pathway analysis provided novel insights into therapies based on the oxidative metabolism and lipid metabolism activity in melanoma with *CCND1* amplification. Recently, energy metabolism in tumor cells has been under growing scrutiny. Oxidative metabolism, especially high levels of ROS, have been associated with induction of tumorigenesis, which involves many elements including DNA damage, and *Ras* and *Myc* oncogenes (51, 52). Accordingly, in our study, we observed the characteristic activation of oxidative metabolism in response to *CCND1* amplification, indicating that treatment strategies blocking oxidative phosphorylation, limiting adaptions to ROS signaling, and increasing antioxidant systems may be effective in melanoma with *CCND1* amplification. We speculate that *CCND1* gene amplification activates metabolism signaling pathways, such as the enhanced oxidative metabolism in cancer cells promotes activation of PI3K-Akt-mTOR signal axis, and then constructs an immune barrier of tumor microenvironment, resulting in resistance to ICIs therapies. This mechanism is the next thing we are going to verify. In addition, our study also identified aberrantly activated lipid metabolism in melanoma with *CCND1* amplification (**Figure 5A**). Prior reports have shown that enhancing fatty acid catabolism improved CD8^+^ T cell antitumor effects (53). Viewed from the perspective of cancer cell biology, recent reports that cancer cells exploit sapient biosynthesis indicate an alternative fatty acid metabolism that can increase cancer plasticity (54). Our data showed an elevated lipid metabolism that may originate from the CD8^+^ T cell and melanoma cells subjected to hypoxia and hypoglycemia. Thus, it is tempting to speculate that targeting lipid metabolism, for instance utilizing a PPAR-α agonist which specifically targeted T cells in the TME (53), to promote fatty acid catabolism by CD8^+^ T cells, or blocking the activated fatty acid metabolism pathway of cancer cells, may be a potential therapeutic option.

In summary, we analyzed data from three different cohorts of patients with melanoma to explore *CCND1* amplification as a distinct genomic subtype. We confirmed that *CCND1* amplification is an unfavorable prognostic factor for patients with melanoma, especially for patients receiving ICIs and who were simultaneously harboring high TMB. We also confirmed the presence of an immunosuppressive TME, down-regulation of angiogenesis-related molecules, and specifically activated metabolism signaling pathways in this melanoma subtype. Understanding the mechanism by which *CCND1* amplification is associated with a poor response to ICIs can provide a basis for developing therapeutic strategies to improve the efficacy of current immunotherapies. Based on our findings, we propose potential therapeutic options such as avoiding ICIs and antiangiogenic monotherapy, while employing CDK4/6 inhibitors alone or in combination with ICIs, and targeting oxidative metabolism and lipid metabolism pathways. We believe the definition of *CCND1* amplification as a unique genomic melanoma subtype, and application of genotype-specific treatments, offers a promising direction for the development of therapeutic strategies for treating melanoma patients with *CCND1* amplification.

## Data Availability Statement

The original contributions presented in the study are included in the article/**Supplementary Material**. Further inquiries can be directed to the corresponding authors.

## Ethics Statement

The studies involving human participants were reviewed and approved by the Ethics Committee of Fujian Provincial Cancer Hospital. The patients/participants provided their written informed consent to participate in this study. The animal study was reviewed and approved by the Institutional Animal Care and Use Committee of Fujian Medical University.

## Author Contributions

JLiu, JLin, XW, XZ carried out the whole research. XG, YH, JX statistically analyzed all the data and graphed. BL help in assays. GC and CC developed the concept. LS and YC obtained the funds and organized the study. All authors read and approved the final manuscript.

## Funding

The work was supported by in part by the National Natural Science Foundation of China (Grant No. U1705282, 32000550), Fujian provincial health and family planning research talent training program (Grant No. 2018-ZQN-13), Natural Science Foundation of Fujian Province (Grant No. 2020J05073), Fujian Provincial Clinical Research Center for Cancer Radiotherapy and Immunotherapy (Grant No.2020Y2012).

## Conflict of Interest

The authors declare that the research was conducted in the absence of any commercial or financial relationships that could be construed as a potential conflict of interest.

## Publisher’s Note

All claims expressed in this article are solely those of the authors and do not necessarily represent those of their affiliated organizations, or those of the publisher, the editors and the reviewers. Any product that may be evaluated in this article, or claim that may be made by its manufacturer, is not guaranteed or endorsed by the publisher.
