# Supplementary material for: CCND1 Amplification Profiling Identifies a Subtype of Melanoma Associated With Poor Survival and an Immunosuppressive Tumor Microenvironment
Source: Front Immunol. 2022 Jul 1;13:725679. doi: 10.3389/fimmu.2022.725679 (PMC9285001; doi:10.3389/fimmu.2022.725679)
Supplement: Supplementary file 1 [file DataSheet_1.pdf]

Supplementary materials

Figure S1. The overlap among CCND1, GAB2, and PAK1 amplification (A) and high amplification (B) in the TCGA-melanoma patients.

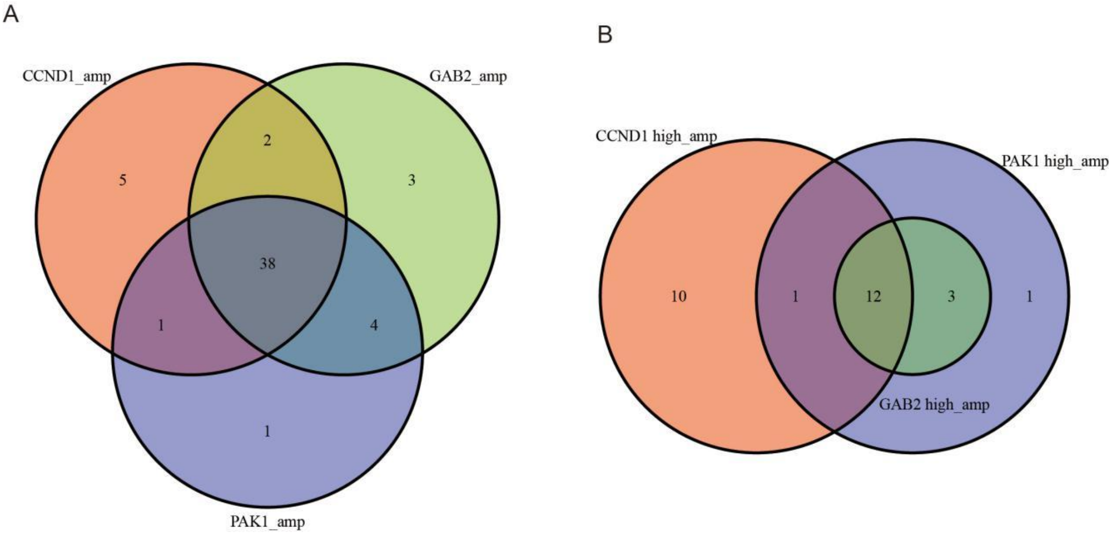

Figure S2: The CCND1 mRNA expression quantified by qPCR.

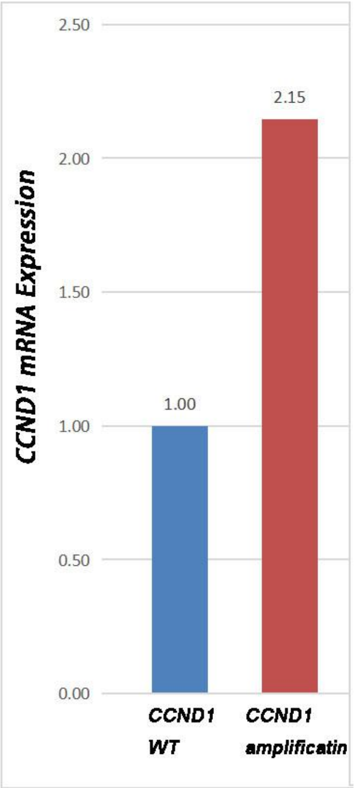

Table S1. Univariate analysis of factors influencing ICIs response.

	Univariate analysis	
	Hazard ratio (95% CI)	P
MSKCC-IO cohort (n=1105)		
CCND1 Amplification (+2) VS. Neutral (0)	1.626 (1.086-2.434)	0.002

FGF3 Amplification (+2) VS. Neutral (0)	1.756 (1.137-2.713)	<0.001
FGF4 Amplification (+2) VS. Neutral (0)	1.756 (1.137-2.713)	<0.001
FGF19 Amplification (+2) VS. Neutral (0)	1.774 (1.153-2.730)	<0.001

Table S2. Characteristics of 14 patients with *CCND1* amplification in the MSKCC-IO cohort

**Characteristics**	***CCND1*-Amplification (*n*=14)**
Sex, n (%)	
Male	11 (78.6)
Female	3 (21.4)
Age (y), n (%)	
≤60	6 (42.9)
>60	8 (57.1)
Normalized mutation count	
Top 20% of each histology	4 (28.6)
Bottom 80% of each histology	10 (71.4)
Drug Class	
PD-1/PD-L1	6 (42.8)
CTLA-4	4 (28.6)
Combination	4 (28.6)
Year of ICI start	
2011-2012	2 (14.3)
2013-2014	2 (14.3)
2015-2017	10 (71.4)

CTLA-4, cytotoxic T-lymphocyte-associated protein 4; ICI, immune checkpoint inhibitors; MSKCC, the Memorial Sloan Kettering Cancer Center; MSKCC-IO, patients in the MSKCC cohort who had received at least one dose of immune checkpoint inhibitor; PD-1, programmed death-1; PD-L1, programmed death ligand-1.

Table S3. Multivariable analysis of factors associated with overall survival in 231 patients in the MSKCC-IO cohort

**Variable**	**OS**		
	P	**Hazard ratio**	**95% CI**
CCND1 copy number alteration	0.006	2.439	1.297-4.587

MSKCC, the Memorial Sloan Kettering Cancer Center; MSKCC-IO, patients in the MSKCC cohort who had received at least one dose of immune checkpoint inhibitor; OS, overall survival.
